# Glutamate Receptor-Mediated Neurotoxicity in a Model of Ethanol Dependence and Withdrawal in Rat Organotypic Hippocampal Slice Cultures

**DOI:** 10.3389/fnins.2018.01053

**Published:** 2019-01-24

**Authors:** Elisabetta Gerace, Elisa Landucci, Daniele Bani, Flavio Moroni, Guido Mannaioni, Domenico E. Pellegrini-Giampietro

**Affiliations:** ^1^Section of Clinical Pharmacology and Oncology, Department of Health Sciences, University of Florence, Florence, Italy; ^2^Section of Pharmacology and Toxicology, Department of Neuroscience, Psychology, Drug Research and Child Health (NeuroFarBa), University of Florence, Florence, Italy; ^3^Research Unit of Histology and Embryology, Section of Anatomy and Histology, Department of Experimental and Clinical Medicine, University of Florence, Florence, Italy

**Keywords:** ethanol withdrawal, glutamate receptors, organotypic hippocampal slices, CA1 injury, neuroprotection

## Abstract

Long-term alcohol use can lead to alterations in brain structure and functions and, in some cases, to neurodegeneration. Several mechanisms have been proposed to explain ethanol (EtOH)-related brain injury. One of the most relevant mechanisms of alcohol-induced neurodegeneration involves glutamatergic transmission, but their exact role is not yet fully understood. We investigated the neurochemical mechanisms underlying the toxicity induced by EtOH dependence and/or withdrawal by exposing rat organotypic hippocampal slices to EtOH (100–300 mM) for 7 days and then incubating the slices in EtOH-free medium for the subsequent 24 h. EtOH withdrawal led to a dose-dependent CA1 pyramidal cell injury, as detected with propidium iodide fluorescence. Electron microscopy of hippocampal slices revealed that not only EtOH withdrawal but also 7 days chronic EtOH exposure elicited signs of apoptotic cell death in CA1 pyramidal cells. These data were supported by electrophysiological recordings of spontaneus Excitatory Post Synaptic Currents (sEPSCs) from CA1 pyramidal cells. The average amplitude of sEPSCs in slices treated with EtOH for 7 days was significantly increased, and even more so during the first 30 min of EtOH withdrawal, suggesting that the initial phase of the neurodegenerative process could be due to an excitotoxic mechanism. We then analyzed the expression levels of presynaptic (vGlut1, vGlut2, CB1 receptor, synaptophysin) and postsynaptic (PSD95, GluN1, GluN2A, GluN2B, GluA1, GluA2, mGluR1 and mGluR5) proteins after 7 days EtOH incubation or after EtOH withdrawal. We found that only GluA1 and mGluR5 expression levels were significantly increased after EtOH withdrawal and, in neuroprotection experiments, we observed that AMPA and mGluR5 antagonists attenuated EtOH withdrawal-induced toxicity. These data suggest that chronic EtOH treatment promotes abnormal synaptic transmission that may lead to CA1 pyramidal cell death after EtOH withdrawal through glutamate receptors and increased excitotoxicity.

## Introduction

Alcohol addiction is a chronic, relapsing brain disease characterized by mental and physical health problems with devastating consequences. The World Health Organization (WHO) estimates that there are about 208 million people with alcoholism worldwide (4.1% of the population over 15 years of age), about 2.3 million people die for a cause alcohol-related and 76.3 million have alcohol use disorders, with social and economic cost, ranging from 1 to 6% of the Gross Domestic Product (GDP) of a country (World Health Organization [WHO], 2014).

Chronic alcohol exposure and alcohol protracted withdrawal causes profound neuroadaptive changes in neuronal excitability and synaptic plasticity that are the consequences of a complex interplay of biological vulnerability, environmental exposure, and developmental factors (Koob and Le Moal, 2008). The efficacies of current pharmacotherapies targeting alcohol dependence are limited also because alcohol affects diverse neurotransmitter systems in the brain (i.e., dopaminergic, GABAergic, glutamatergic and serotonergic systems) (LeMarquand et al., 1994; Charlet et al., 2013). Importantly, following long-term alcohol consumption, the brain compensates the depressant effects of alcohol to maintain homeostasis between inhibitory and excitatory neurotransmission by increasing excitatory activity and reducing inhibitory activity (Nam et al., 2012; Koob, 2013; Tabakoff and Hoffman, 2013).

Glutamate plays a pivotal role in drug addiction, drug self-administration, reward-related processes and relapse (Kalivas and Volkow, 2005; Gass and Olive, 2008). Increased glutamatergic neurotransmission and hyper-excitability during withdrawal and abstinence are associated with an increased risk for relapse (Tsai and Coyle, 1998), and changes in the glutamatergic system after chronic alcohol intake has been largely described (Nagy et al., 2005; Krupitsky et al., 2007) and numerous reports have demonstrated the functional relationship observed in EtOH dependent and withdrawn animals and the glutamatergic transmission (Läck et al., 2007; Marty and Spigelman, 2012; Gerace et al., 2016). Elevated levels of glutamate has been measured in the medial prefrontal cortex of alcoholic patients and alcohol-dependent rats during acute withdrawal (Hermann et al., 2012). NMDA receptor alterations and functioning have been described to contribute to neuronal excitation and neurotoxicity during EtOH withdrawal (Butler et al., 2013), whereas AMPA receptors have been shown to play a key role in the regulation of reinforcement and the reward process of EtOH (Stuber et al., 2008; Wang et al., 2012). Preclinical research suggests the important role of metabotropic glutamate subtype 5 (mGlu5) receptor in EtOH drinking (Cozzoli et al., 2009, 2012; Besheer et al., 2010; Sinclair et al., 2012) and in the memory formation responsible for the chronic relapsing nature of alcohol abuse (Obara et al., 2009). Nevertheless, the exact mechanisms by which ethanol exerts its toxic effects through glutamatergic transmission are still unknown.

In the present study, we investigated the neurochemical mechanisms underlying the toxicity induced by EtOH in organotypic hippocampal slice models of dependence and/or withdrawal. In particular, we investigated the role of ionotropic and metabotropic glutamate receptors in mediating toxicity induced by the chronic exposure to EtOH and following EtOH withdrawal.

## Materials and Methods

Experiments and animal use procedures were in accordance with the National Institutes of Health Guide for the Care and Use of Laboratory Animals (NIH Publications No. 80-23, revised 1996). The experimental protocols were approved by the Animal Care Committee of the Department of Health Sciences, section of Pharmacology, University of Florence, in compliance with the European Convention for the Protection of Vertebrate Animals used for Experimental and Other Scientific Purposes (ETS no. 123), the Department of Neuroscience, Psychology, Drug Research and Child Health (NeuroFarBa) (ETS no. 176) and the European Communities Council Directive of 24 November 1986 (86/609/EEC). The authors further attest that all efforts were made to minimize the number of animals used and their suffering.

### Materials

Propidium iodide (PI) was purchased from Sigma (St Louis, MO, United States). Tissue culture reagents were obtained from Gibco-BRL (San Giuliano Milanese, MI, Italy) and Sigma (St Louis, MO, United States). 2,3-Dioxo-6-nitro-1,2,3,4-tetrahydrobenzo[*f*]quinoxaline-7-sulfonamide disodium salt (NBQX) and 2-Methyl-6-(phenylethynyl)pyridine (MPEP) hydrochloride were purchased from Tocris (Bristol, United Kingdom).

### Preparation of Rat Organotypic Hippocampal Slice Cultures

Organotypic hippocampal slice cultures were prepared as previously reported Gerace et al., 2012a, 2015, Landucci et al., 2018). Briefly, hippocampi were removed from the brains of 7- to 9-day old Wistar rat pups (Harlan, MI, Italy), transverse slices (420 μm) were prepared using a McIlwain tissue chopper and then transferred onto 30 mm diameter semiporous membranes inserts (Millicell-CM PICM03050; Millipore, Italy), which were placed in six well tissue culture plates containing 1.2 ml medium per well. The culture medium consisted of 50% Eagle’s minimal essential medium, 25% heat-inactivated horse serum, 25% Hanks’ balanced salt solution, 5 mg/ml glucose, 2 mM L-glutamine, and 3.75 mg/ml amphotericin B. Slices were maintained at 37 °C in an incubator in atmosphere of humidified air and 5% CO_2_ for 10 days. Before experiments all slices were screened for viability by incubating them for 30 min with PI (5 μg/ml); slices displaying signs of neurodegeneration were discarded from the study.

### Ethanol Exposure and Drug Treatment in Organotypic Hippocampal Slices

Rat organotypic hippocampal slice cultures were exposed for 7days to 100, 150, or 300 mM of EtOH (corresponding respectively to 4.6, 6.9, or 13.8 g/l) after 10 days of culture *in vitro* as described in Gerace et al. (2016). The medium was changed every day adding ethanol to the fresh culture medium. After 7 days of EtOH treatment, some of the slices were incubated in EtOH -free medium or in ethanol-free medium plus the AMPA antagonist NBQX and/or the metabotropic Glu5 antagonist MPEP for 24 h before they were assessed for neuronal injury using PI fluorescence. As discussed (Gerace et al., 2012b, 2016; Landucci et al., 2016), the concentrations of drugs used in organotypic hippocampal slice experiments are generally somewhat higher than those expected from their Kd values and those used in cell cultures. This is due to the fact that they diffuse slowly through the thickness of brain tissue *in vitro*. Moreover, in the case of organotypic slices statically cultured on semiporous membrane inserts at the interface between culture medium and gas atmosphere, the concentrations of drugs need to be further increased because only the bottom of the slice is exposed to the bathing medium to which the drug is added and only a fraction of the drug diffuses across the membrane and reaches the tissue.

### Assessment of CA1 Pyramidal Cell Injury

Propidium iodide (5 μg/ml) was added to the medium either at the end of the 7-day EtOH incubation period or 24 h after it was removed from the medium. Thirty minutes later, fluorescence was viewed using an inverted fluorescence microscope (Olympus IX-50; Solent Scientific, Segensworth, United Kingdom) equipped with a xenon-arc lamp, a low-power objective (4X) and a rhodamine filter. Images were digitized using a video image obtained by a CCD camera (Diagnostic Instruments Inc., Sterling Heights, MI, United States) controlled by software (InCyt Im1^TM^; Intracellular Imaging Inc., Cincinnati, OH, United States) and subsequently analyzed using the Image-Pro Plus morphometric analysis software (Media Cybernetics, Silver Spring, MD, United States). In order to quantify cell death, the CA1 hippocampal subfield was identified and encompassed in a frame using the drawing function in the image software (ImageJ; NIH, Bethesda, MD, United States) and the optical density of PI fluorescence was detected. There was a linear correlation between CA1 PI fluorescence and the number of injured CA1 pyramidal cells as detected by morphological criteria (Pellegrini-Giampietro et al., 1999).

### Electron Microscopy in CA1 Region of Hippocampal Slices

At the end of the experiments the slices were washed with cold 0.01 M phosphate-buffered saline, pH 7.4 and were directly fixed in cold 4% glutaraldehyde in 0.1 M sodium cacodylate buffer (pH 7.4) overnight at 4°C and post-fixed in cold 1% osmium tetroxide in 0.1 M phosphate buffer (pH 7.4) for 1 h at room temperature as described in Gerace et al. (2016). The samples were dehydrated in graded acetone, passed through propylene oxide, and embedded in Epon 812. Ultrathin sections were stained with uranyl acetate and alkaline bismuth subnitrate and examined under a JEM 1010 electron microscope (Jeol, Tokyo, Japan) at 80 kV.

### Electrophysiological Recordings in Organotypic Hippocampal Slices

Whole-cell voltage-clamp recordings were performed as described in Gerace et al. (2016). Briefly, inward currents were recorded in CA1 pyramidal cells from organotypic hippocampal slices chronically treated with 150 mM of EtOH or under control conditions. Slices were removed from the culture insert and placed into a recording bath submerged with ice-cold artificial cerebrospinal fluid (ACSF) containing (in mM): EtOH 150, NaCl 130, KCl 3.5, Na_2_H_2_PO_4_ 3, NaHCO_3_, glucose, MgCl_2_ 1.5 and CaCl_2_ 1.5 at pH 7.4 and oxygenated with 95%O_2_/5%CO_2_).

Recording microelectrodes were prepared from borosilicate glass (WPI Inc; Sarasota, FL, United States) by a Narishige Instruments micropipette puller (Tujunga, CA, United States) (resistance ranging from 3 to 5 MΩ) and filled with internal solution of the following composition (in mM concentrations): K-gluconate 142.5, potassium methylsulfate 20, NaCl 8, Hepes 10, EGTA 0.1, MgATP 2, and GTP 0.2. The pH of the internal solution was adjusted to 7.2 with KOH and the osmolarity was adjusted to 300 mOsm with H_2_O and sucrose. After establishing a GIGA seal a whole-cell configuration was achieved by rupturing the membrane. Recordings were done in voltage clamp configuration (holding potentials -60 mV) using a Multiclamp preamplifier (Axon Instruments; Foster City, CA, United States) and filtered at 5 kHz. All the data were acquired, stored and analyzed on a PC using the pCLAMP (Axon Instruments, Foster City, CA, United States) and GraphPad softwares. Traces were filtered by a digital Gaussian filter (Clampfit facility, low pass, 200 Hz). The frequency and peak amplitude of detected events were analyzed using Mini Analysis Program (Synaptosoft Inc., Fort Lee, NJ, United States)^1^. The AMPA receptor blocker NBQX (10 μM) was added at the end of each experiment to verify that the spontaneous excitatory post synaptic currents were AMPA receptor-mediated.

### Western Blot Analysis

Cultured slices were washed with cold 0.01 M phosphate-buffered saline, pH 7.4 and 8 slices/sample were gently transferred and dissolved in a tube containing 1% SDS. Total protein levels were quantified using the Pierce (Rockford, IL, United States) BCA (bicinchoninic acid) Protein Assay. Forty μg of proteins were resolved by electrophoresis on a 8 or 12% SDS-polyacrylamide gel and transferred onto nitrocellulose membranes using the transblot TURBO (Bio-Rad, Hercules, CA, United States). Blots were probed overnight at 4°C with the polyclonal rabbit vGluT1, vGluT2, GluN2A, GluA1, metabotropic Glu1, PSD95, polyclonal goat GluA2, GluN1 and monoclonal mouse metabotropic Glu5 antibodies (Millipore, Italy), synaptophysin and GluN2B antibodies (Thermo Scientific, Rockford, IL, United States), C-terminal rabbit anti-CB1 polyclonal antibody (generously provided by Dr. Ken Mackie, University of Washington, Seattle, WA, United States), all diluted 1:1000 (Gerace et al., 2012b, 2016). Immunodetection was performed with secondary antibodies [1:2000 anti-mouse or anti-rabbit IgG from donkey or anti goat (Amersham Biosciences, United Kingdom) conjugated to horseradish peroxidase. The reactive bands were detected using chemiluminescence (ECLplus; Euroclone, Padova, Italy). Quantitative analysis was performed using the QuantityOne analysis software (Bio-Rad, Hercules, CA, United States).

### Statistical Analysis

Data are presented as means ± SEM of *n* experiments. Statistical significance of differences between PI fluorescence intensities or Western blot optical densities was evaluated by performing one-way ANOVA followed by Dunnet’s test for multiple comparisons or by the Kolmogorov–Smirnov test (sEPSC recordings). All statistical calculations were performed using GRAPH-PAD PRISM v. 5 for Windows (GraphPad Software, San Diego, CA, United States). A probability value (*P*) of < 0.05 was considered significant.

## Results

**FIGURE 1:**
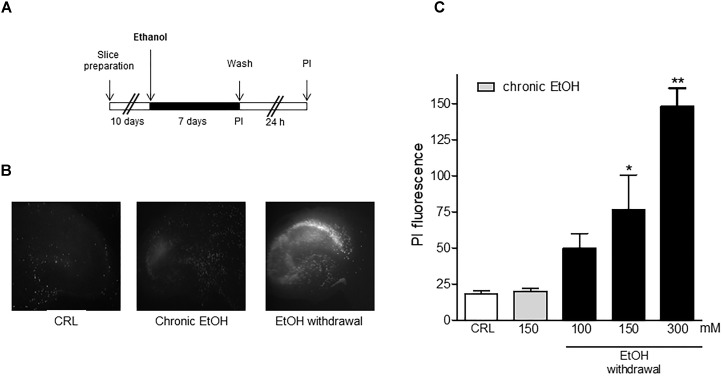
Ethanol withdrawal induces cell death in mature organotypic hippocampal slices. **(A)** Experimental protocol showing hippocampal slices cultured for 10 days *in vitro* and exposed to 100, 150, 300 mM of ethanol (corresponding to 4.6, 6.9, or 13.8 g/l of plasmatic EtOH concentration in humans) for 7 days. At the end of this period (Chronic ethanol), ethanol was removed from the medium. 24 h later (Withdrawal) the fluorescent dye propidium iodide (PI) was added to the medium to assess neuronal injury. **(B)** Mature hippocampal slices, photographed under fluorescence optics, displaying background levels of fluorescence under control or chronic ethanol condition (150 mM) and an intense PI labeling after ethanol withdrawal (150 mM), showing a selective CA1 pyramidal cell injury. **(C)** Cell injury was assessed using the fluorescent dye propidium iodide at the end of the chronic EtOH treatment (150 mM) and after 24 h of EtOH withdrawal. Quantitative data are expressed as CA1 PI fluorescence. Values represent the mean ± SEM of 5 experiments in ethanol withdrawal condition. ^∗^*p* < 0.05 and ^∗∗^*p* < 0.01 vs. basal PI fluorescence (ANOVA + Dunnet’s test).

Figure 1 shows that organotypic hippocampal slices exposed to chronic EtOH for 7 days and then incubated in EtOH -free-medium for the subsequent 24 h displayed a selective and dose-dependent CA1 pyramidal necrotic cell death. The concentrations of EtOH decreased within 24 h from 127 mM (5.8 g/l) to 32 mM (1.5 g/l), which is similar to the blood alcohol concentration detected in the blood of alcoholics in the emergency room as shown in Gerace et al. (2016).

**FIGURE 2:**
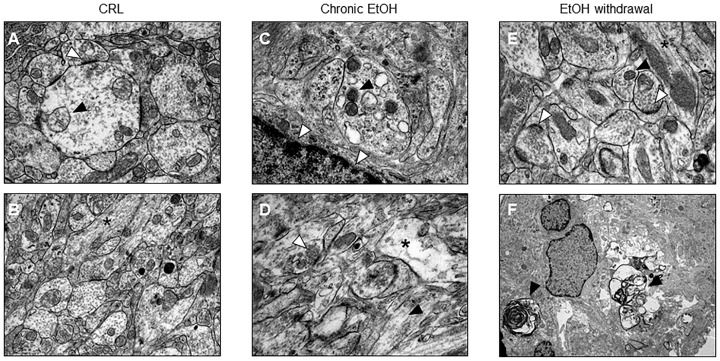
Electron microscopic evidence for apoptotic cell death in organotypic hippocampal slices. **(A,B)** Control healthy CA1 pyramidal cells showing healthy mithocondria (black arrow) and synapses rich in vescicles (white arrow) **(A)** and in longitudinally aligned microtubules in the neuronal processes (black asterisk) **(B)**. **(C,D)** CA1 pyramidal cells from chronic ethanol slices display suffering mithocondria (black arrow), accumulation of lipofuscins (white arrows) **(C)** and intercellular empty spaces (black asterisk), but regular synapses (white arrow) and in longitudinally aligned microtubules in the neuronal processes (black arrow) **(D)**. **(E,F)** Electron microscope morphology in control organotypic slices showing normal synapses with presynaptic vescicles (white arrows) and in longitudinally aligned microtubules in the neuronal processes (black asterisk) **(E)** and clear signs of apoptosis (black arrows) **(F)**.

Electron microscopy confirmed what has been observed with PI fluorescence and shows that after EtOH withdrawal the slices undergo to neuronal death (Figures 2E,F). Moreover, CA1 pyramidal cell bodies exposed to EtOH for 7 days displayed suffering mithocondria, accumulation of lipofuscins and intercellular empty spaces (Figure 2C,D) as compared to control organotypic slices (Figures 2A,B), suggesting that not only withdrawal but also 7 days chronic EtOH exposure elicited signs of apoptotic cell death in CA1 pyramidal cells.

**FIGURE 3:**
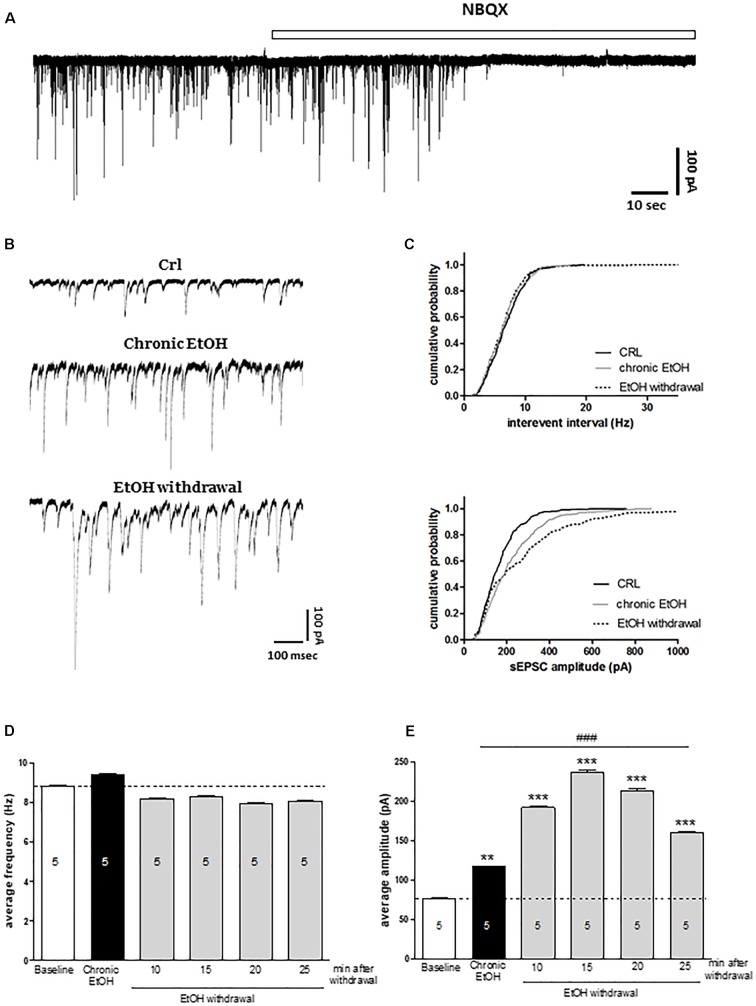
Chronic ethanol and ethanol withdrawal increase the amplitude of sEPSCs in CA1 pyramidal cells of organotypic hippocampal slices. Experiments were conducted as described in Figure 1A. **(A)** Example trace showing the effect of the AMPA antagonist NBQX on sEPSCs recorded in CA1 pyramidal neurons of control organotypic hippocampal slices in whole-cell voltage clamp configuration. **(B)** Representative traces showing sEPSCs recorded in CA1 pyramidal neurons of organotypic hippocampal slices in whole-cell voltage clamp configuration under control, chronic ethanol incubation and ethanol withdrawal conditions. **(C)** Cumulative probability plots demonstrating the effect of EtOH withdrawal on sEPSC interevent interval and amplitude. EtOH withdrawal caused a significant shift in the amplitude distributions, indicating a significant increase in the amplitude of sEPSCs [Kolmogorov–Smirnov (K–S) statistic CRL vs. chronic EtOH and CRL vs. EtOH withdrawal *p* < 0.0001]. On the contrary, the cumulative probability plots on sEPSC interevent interval did not show any significant changing (K–S statistic CRL vs. chronic EtOH *p* = 0.977, CRL vs. EtOH withdrawal *p* = 0.462). Bar graphs displaying the values of sEPSC frequency (Hz) **(D)** and amplitude (pA) **(E)** during the time course of the last 5 min of chronic ethanol treatment and the first 30 min of ethanol withdrawal. Bars represent the mean of at least 5 experiments. ^∗∗∗^*p* < 0.001 vs. CRL and ^∗∗^*p* < 0.01, ^###^*p* < 0.01 vs. chronic EtOH (ANOVA + Tukey’s *w* test). The number of cells is indicated on the bar graphs.

In order to understand the molecular mechanisms underlying the toxicity induced by EtOH withdrawal in organotypic hippocampal slices, we performed electrophysiological experiments by using the patch clamp technique. We recorded sEPSCs from CA1 pyramidal cells exposed to EtOH for 7 days (as shown in Figure 3B) and during the first 30 min of EtOH withdrawal. Figure 3A represents an example trace of the effect of the AMPA antagonist NBQX on sEPSCs in control slice, showing that under our experimental conditions sEPSCs are mainly if not exclusively AMPA-mediated. We did not observe differences in the holding current between control slices and slices chronically exposed to EtOH for 7 days (100 ± 44 pA, *n* = 5 vs. 140 ± 32.6 pA, *n* = 5, respectively). Figure 3B show examples of sEPSCs recorded under control conditions, after chronic incubation with EtOH for 7-days and after 15 min of EtOH withdrawal. Our results show that chronic EtOH exposure increases the amplitude but not the frequency of sEPSCs (Figure 3). Interestingly, EtOH withdrawal further increases sEPSCs amplitude without affecting sEPSCs frequency compared to chronic EtOH exposure, thus indicating that glutamatergic transmission is involved in both EtOH dependence and withdrawal processes (Figure 3). Kolmogorov–Smirnov analyses confirms that both the chronic incubation with EtOH or its withdrawal produced a rightward shift of the cumulative probability distribution of sEPSC amplitude (Figure 3B, lower panel).

**FIGURE 4:**
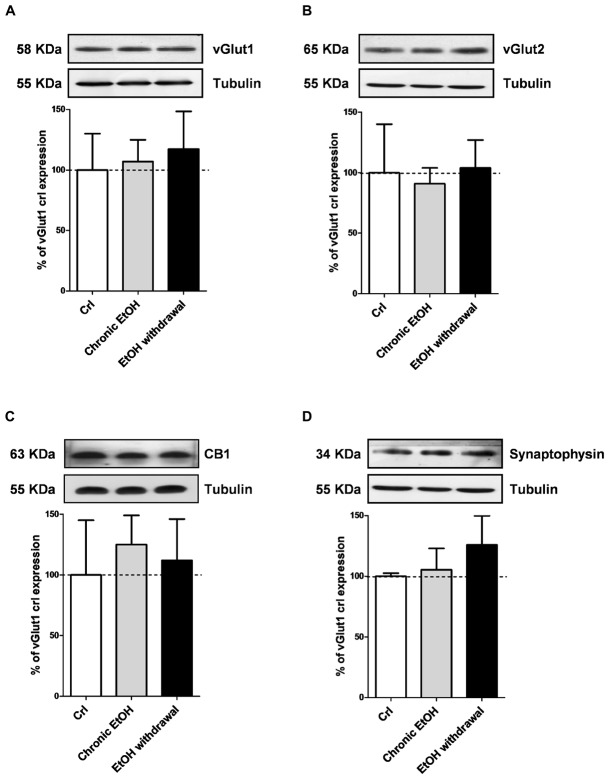
Chronic ethanol and ethanol withdrawal do not modify the expression of presynaptic proteins in organotypic hippocampal slices. Experiments were conducted as described in Figure 1A. At the end of the experiments hippocampal slices were lysed and processed for Western blot. *Top:* representative Western blots using antibodies against the pre-synaptic proteins vGlut1, vGlut2, CB1 and synaptophysin **(A–D)**. The numbers on the left indicate the position of the molecular mass markers (kDa). Tubulin was used as loading control. *Bottom:* quantitative analysis of immunoreactive bands, showing that chronic ethanol nor ethanol withdrawal modify the expression of vGlut1, vGlut2, CB1 and synaptophysin. Data are expressed as percentage of control. Bars represent the mean ± SEM of at least three experiments. ^∗^*P* < 0.05 vs. CRL (ANOVA + Dunnet’s test).

**FIGURE 5:**
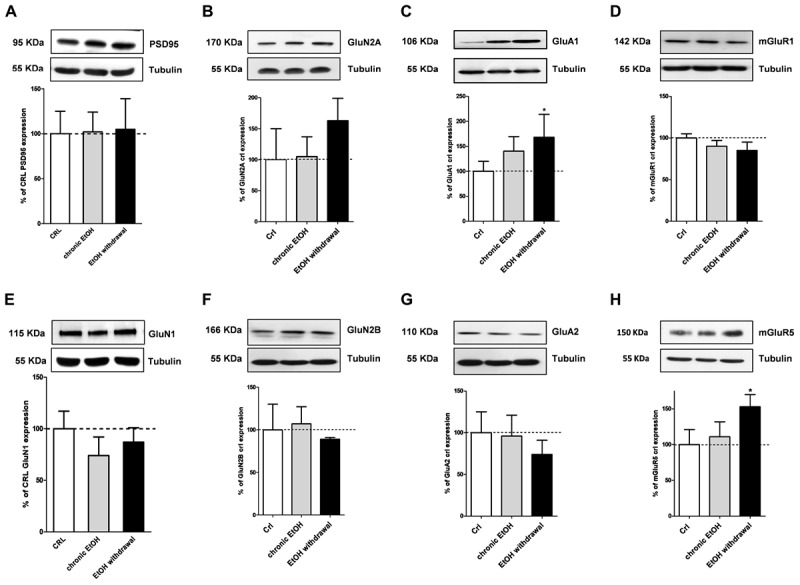
Ethanol withdrawal increases the expression of the GluA1 AMPA subunit and of mGluR5 proteins in organotypic hippocampal slices. Experiments were conducted as described in Figure 1A. At the end of the experiments hippocampal slices were lysed and processed for Western blot. *Top:* representative Western blots using antibodies against the post-synaptic proteins PSD95, GluN1, GluN2A, GluN2B, GluA1, GluA2, mGluR1, and mGluR5 **(A–H)**. The numbers on the left indicate the position of the molecular mass markers (kDa). Tubulin was used as loading control. *Bottom:* quantitative analysis of immunoreactive bands, showing that ethanol withdrawal increases the expression of the AMPA subunit GluA1 and of metabotropic glutamate receptors mGluR5. Data are expressed as percentage of control. Bars represent the mean ± SEM of at least three experiments. ^∗^*P* < 0.05 vs. CRL (ANOVA + Dunnet’s test).

We then performed Western blotting analysis in organotypic hippocampal slices exposed to EtOH using antibodies directed against specific pre- and post-synaptic proteins (Figures 4, 5, respectively). Chronic incubation with EtOH for 7 days and its removal from the medium did not change the expression of the presynaptic proteins vGluT1, vGluT2, CB1 receptors and synaptophysin (Figure 4). On the other hand, at the postsynaptic level, EtOH withdrawal induced an increased expression of the AMPA subunit GluA1 and of the metabotropic Glu5 receptors, while chronic EtOH exposure showed a trend toward an increased expression without reaching a statistical significance (Figures 5B,F) of the same proteins. No changes for NMDA subunits (GluN1, GluN2A, and GluN2B) nor for the scaffold protein PSD95, GluA2 AMPA subunit and mGluR1 receptors were observed at the postsynaptic level (Figure 5) in both chronic EtOH exposure and EtOH withdrawal.

**FIGURE 6:**
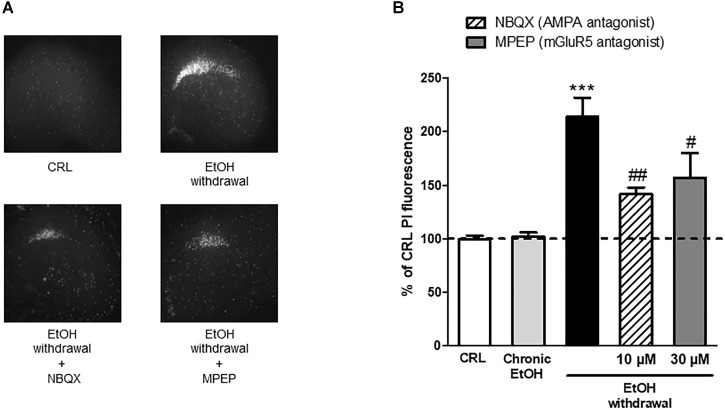
AMPAand mGluR5 antagonists attenuate the neurotoxicity induced by ethanol withdrawal in organotypic hippocampal slices. **(A)**: Hippocampal slices, photographed under fluorescence optics, displaying background levels of fluorescence under control conditions, an intense PI labeling in the CA1 subregion 24 h after ethanol withdrawal, and a reduction of CA1 PI fluorescence when incubated with 10 μM NBQX and 30 μM of MPEP during the 24 h withdrawal period. **(B):** Quantitative analysis of CA1 region expressed as percentage of CRL PI fluorescence. Incubation with the AMPA antagonist NBQX and with the selective mGluR5 antagonist MPEP significantly attenuated ethanol-withdrawal-induced injury. Values represent the mean ± SEM of at least five experiments. ^∗∗∗^*P* < 0.001 vs. CRL and ^#^ < 0.05 vs. ethanol withdrawal alone and ^##^ < 0.01 vs. ethanol withdrawal alone (ANOVA + Tukey’s *w* test).

These results propose a central role for AMPA and metabotropic Glu5 receptors in EtOH -induced CA1 toxicity and suggest AMPA and mGlu5 as possible therapeutic targets. Therefore, we used a therapeutic approach *in vitro* by exposing the slices to the AMPA competitive antagonist NBQX and to the selective mGluR5 non-competitive antagonist MPEP during the 24 h EtOH withdrawal period. We observed that both these agents were able to significantly attenuate EtOH withdrawal-induced neurotoxicity in the CA1 hippocampal subregion (34 ± 2.7% and 26.7 ± 10.7% NBQX and MPEP, respectively) (Figure 6) confirming that AMPA channels and mGlu5 receptors are implicated in EtOH withdrawal injury.

## Discussion

In the present study, we investigated the neurochemical mechanisms underlying the toxicity induced by EtOH in organotypic hippocampal slice models of EtOH dependence and/or withdrawal. Rat organotypic hippocampal slices (10 DIV) were exposed for 7 days to concentrations of EtOH ranging between 100 and 300 mM (corresponding to 4.6 and 13.8 g/l respectively). As reported in Gerace et al. (2016), the actual EtOH concentration measured in our culture media decreased within 24 h from 127 mM (5.8 g/l) to 32 mM (1.5 g/l), which reproduces the blood alcohol concentration oscillations typical of a frequent drinker and is compatible with the high EtOH peak concentrations (between 3 and 4 g/l) detected in the blood of alcoholics in the emergency room. The slices were then incubated in EtOH-free medium for the subsequent 24 h to mimic EtOH withdrawal. Under these conditions, we observed that EtOH withdrawal led to a dose-dependent CA1 pyramidal necrotic cell death as detected by PI fluorescence and confirmed by electron microscopy, revealing clear signs of injury in CA1 pyramidal cell bodies. Our results are in agreement with previous studies showing neuronal damage and permanent impairment in synaptic transmission of hippocampal neurons (Prendergast et al., 2000; Harris et al., 2003; Self et al., 2005).

Interestingly, a previous paper from our laboratory shows that, under the same experimental conditions, the deprivation of EtOH is not toxic in slices cultured for only 2 DIV, suggesting that slices that have not reached complete maturation *in vitro* may respond to prolonged EtOH incubation in a different manner as compared to more mature slices, resulting in greater resistance to EtOH toxicity upon its removal (Gerace et al., 2016). The differential age-dependent effects of EtOH were highlighted also by electron microscopy, which revealed, despite the lack of toxicity observed with PI, a clear disorganization of microtubuli in neural processes after chronic EtOH exposure in immature slices (Gerace et al., 2016). On the contrary, microtubuli alignment was observed in mature slices chronically exposed to EtOH in the same CA1 neurons, but also ultrastructural signs of neuronal suffering like distressed mitochondria, accumulation of lipofuscins and intercellular empty spaces as compared to control slices. These results suggest that not only withdrawal but also chronic EtOH exposure may elicit signs of apoptotic cell death in CA1 pyramidal cells.

These data are supported by our electrophysiological recordings of AMPA-mediated sEPSCs from CA1 pyramidal cells after 7 days of EtOH exposure and during the first 30 min of EtOH removal, which revealed a significant increase in the average amplitude but not in the frequency of sEPSCs that was induced not only by EtOH withdrawal but was also present in slices chronically exposed to EtOH, thus suggesting that glutamatergic post-synaptic transmission may be enhanced both during EtOH dependence and following withdrawal. Several reports have demonstrated that glutamatergic transmission is involved in EtOH dependence and withdrawal (Läck et al., 2007; Marty and Spigelman, 2012; Gerace et al., 2016). For example, elevated levels of glutamate have been measured in the medial prefrontal cortex of alcoholic patients and in nucleus accumbens of alcohol-dependent rats during acute withdrawal (Hermann et al., 2012; Pati et al., 2016). Our findings strongly suggest that incubation with EtOH and/or its removal markedly impair the structure and function of the excitatory post-synaptic compartments. On the contrary, in immature organotypic hippocampal slices EtOH induced a decrease in sEPSC frequency and in the expression of specific presynaptic proteins, thus suggesting an impairment of the structure and function of the excitatory pre-synaptic compartments (Gerace et al., 2016).

**FIGURE 7:**
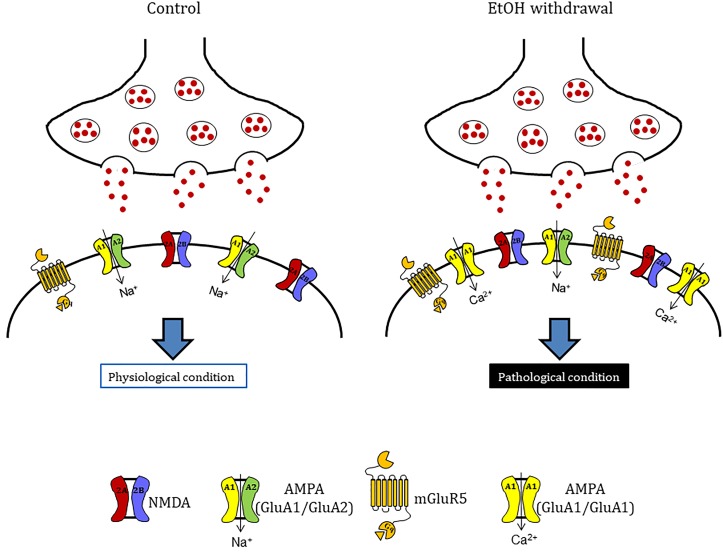
Hypothetical model to explain mechanisms underlying ethanol withdrawal toxicity. Ethanol withdrawal potentiates the excitatory synaptic transmission that lead to CA1 injury of hippocampus *in vitro.* These alterations are probability due to the increase of the expression of the glutamate receptors AMPA (specifically the GluA1 AMPA subunit) and mGluR5. These evidences suggest that after ethanol withdrawal there is a shift in AMPAR subunit composition with the insertion of calcium permeable AMPA receptors in organotypic hippocampal slices.

Moreover, data obtained by Western blotting showing that GluA1 and metabotropic Glu5 receptor expression levels were significantly increased in slices following chronic EtOH incubation and its withdrawal from the medium. On the contrary, no changes in the expression of a number of presynaptic proteins (vGlut1, vGlut2, CB1, and synaptophysin) nor of the scaffold protein PSD95, the NMDA subunits GluN1, GluN2A and GluN2B, the AMPA subunit GluA2 or mGlu1 receptors were observed. Despite NMDA receptor alterations have been described to contribute to neuronal excitation and neurotoxicity following EtOH withdrawal (Butler et al., 2013), we failed to observe any significant change in the expression of its subunits GluN1, GluN2A, and GluN2B under our experimental conditions. Rather, our findings show that following chronic EtOH incubation and its withdrawal there is an increase in AMPA-mediated sEPSCs and in the expression of the GluA1 AMPA subunit and mGlu5 receptors (Figure 7), in agreement with reports demonstrating the implication of AMPA and mGlu5 receptors in various models of EtOH dependence and withdrawal. For example Varodayan et al. (2018) have shown that AMPA receptor-mediated excitatory transmission is enhanced in the medial prefrontal cortex of mice after chronic exposure to EtOH followed by 1 week of withdrawal. Similarly Li et al. (2017) have shown that the amplitude of AMPA EPSCs and GluA1 (S831) phosphorylation, that plays a crucial role in increasing AMPAR activity in various regions of the brain (Malinow, 2003), were increased in neurons of the lateral habenula after 24 h of withdrawal from chronic voluntary EtOH drinking. Consistent with these findings, AMPARs have been shown to play a key role in the regulation of reinforcement and reward processes of EtOH (Stuber et al., 2008; Wang et al., 2012), presumably via an EtOH-dependent increase in the surface concentration of GluA1 subunits in the striatum. As for mGlu5 receptors, a significant increase in its gene expression was observed in the amygdala and in the nucleus accumbens shell of EtOH-withdrawn rats (Kumar et al., 2016; Lee et al., 2018). Moreover, preclinical research suggests an important role of this receptor subtype in EtOH drinking (Cozzoli et al., 2009, 2012; Besheer et al., 2010; Sinclair et al., 2012) and in memory formation, which appears to be responsible for the chronic relapsing nature of alcohol abuse (Obara et al., 2009). In fact, mGlu5 has been implicated in cognition (e.g., memory and learning), novelty seeking behavior, and compulsivity, all factors that may be important in the susceptibility to drug addiction (Leurquin-Sterk et al., 2018).

We observed that both the AMPA competitive antagonist NBQX and the selective mGluR5 non-competitive antagonist MPEP were able to significantly attenuate EtOH withdrawal-induced neurotoxicity in the CA1 hippocampal sub-region, confirming the central role of AMPA channels and mGlu5 receptors in EtOH withdrawal hippocampal injury. The present findings support the results from reports showing that AMPA and mGlu5 receptor antagonists are involved in EtOH-withdrawal-related behaviors. For example, pharmacological inhibition of AMPA receptors reduces EtOH consumption in rats (Li et al., 2017) and the systemic administration of mGlu5 antagonists attenuate cue-evoked reinstatement of ethanol-seeking behavior in rodents (Bäckström and Hyytiä, 2004; Adams et al., 2008; Hodge et al., 2008; Sinclair et al., 2012). Similarly, studies with mGlu5 negative allosteric modulators performed in animal models of addiction have shown a reduction in the self-administration of cocaine, ethanol, and nicotine (Mihov and Hasler, 2016). Finally, our Western blot results show an increase in the expression of GluA1 but not GluA2 AMPA subunits in hippocampal slices following EtOH withdrawal, suggesting a shift in AMPA receptor subunit composition, from GluA2-containing to GluA2-lacking calcium-permeable channels that could explain the CA1 injury observed in our slices following EtOH-withdrawal. An increase in GluA2-lacking calcium-permeable AMPA channels has been shown to promote cell death of hippocampal CA1 pyramidal neurons in models of toxicity (Anzai et al., 2003; Gerace et al., 2014) and in pathological conditions including global ischemia (Pellegrini-Giampietro et al., 1997) and amyotrophic lateral sclerosis (Hideyama and Kwak, 2011). Calcium-permeable AMPA receptors have also been demonstrated to play an important role in addiction models (Mameli et al., 2011; Pascoli et al., 2014): for example, it was recently shown that cocaine-induced potentiation of VTA synapses is mediated by the insertion of calcium permeable AMPA receptors to the synaptic membranes (Mills et al., 2017).

## Conclusion

Our data suggest that chronic EtOH treatment promotes abnormal synaptic transmission that may lead to CA1 pyramidal cell death after EtOH withdrawal through glutamate receptors and increased excitotoxicity. In addition, we propose AMPA and mGlu5 receptors as two candidate targets for new therapeutic interventions for alcohol addiction.

## Author Contributions

EG designed the research studies, conducted the experiments, acquired and analyzed the data, and wrote the manuscript. EL and DB conducted the experiments and acquired and analyzed the data. FM designed the research studies and edited the manuscript. GM designed the research studies, provided reagents, and edited the manuscript. DP-G designed the research studies, provided reagents, and wrote the manuscript.

## Conflict of Interest Statement

The authors declare that the research was conducted in the absence of any commercial or financial relationships that could be construed as a potential conflict of interest.

## Footnotes

1 www.synaptosoft.com
